# Pepsin Preparations

**Published:** 1883-08

**Authors:** Jos. G. Richardson

**Affiliations:** Professor of Hygiene in the University of Pennsylvania. Philadelphia


### Pepsin Preparations.

Eds. Med. and Surg. Reporter :—I have this day tested a specimen of Dr. Jensen's so-called "Crystal Pepsin," with the following results, which may be of interest to your readers:

*Experiment* 1. One-quarter of a grain of Jensen's Pepsin dissolved in f. oz. iss. of water mixed with f. oz. ij. of water containing 9 drops of hydrochloric acid, and kept at a heat varying from 100° to 110° F., dissolved 125 grains of hard-boiled white of egg in two hours.

*Experiment* 2 was an exact copy of the first, except that solution was effected in two and a quarter hours.

*Experiment* 3. One-quarter of a grain of the same pepsin, under like conditions, dissolved 150 grains, or 600 times its weight, of coagulated albumen in about three hours—this amount of albumen being rather more than half that usually found in an egg of average size.

*Experiment* 4. One-quarter of a grain of the pepsin similarly tested with 200 grains of boiled white of egg, left a small residue (estimated to weigh 20 or 25 grains) at the end of three and a half hours, when the experiment was interrupted. It was therefore probably capable under these circumstances of dissolving at least 700 times its own weight of freshly coagulated albumen.

The solvent power of this pepsin is thus shown in these investigations of mine to be not less than twelve times as great as that of the "pepsinum saccharatum" (U. S. Pharm., 1880), and hence this method of preparing pepsin unquestionably places within the reach of physicians a vastly improved means for aiding the stomach digestion of nitrogenous foods.

JOS. G. RICHARDSON, M.D.,

Professor of Hygiene in the University of Pennsylvania.

*Philadelphia, May* 16.

